# Opportunities and Challenges for In Silico Drug Discovery at Delta Opioid Receptors

**DOI:** 10.3390/ph15070873

**Published:** 2022-07-15

**Authors:** Yazan J. Meqbil, Richard M. van Rijn

**Affiliations:** 1 Department of Medicinal Chemistry and Molecular Pharmacology, Computational Interdisciplinary Graduate Program, Purdue University, West Lafayette, IN 47907, USA; ymeqbil@purdue.edu; 2 Department of Medicinal Chemistry and Molecular Pharmacology, Purdue Institute for Drug Discovery, Purdue Institute for Neuroscience, Purdue University, West Lafayette, IN 47907, USA; 3 Septerna Inc., South San Francisco, CA 94080, USA

**Keywords:** mutagenesis, artificial intelligence, computer-aided drug design, molecular dynamic simulation, biased signaling, G protein-coupled receptor

## Abstract

The delta opioid receptor is a Gi-protein-coupled receptor (GPCR) with a broad expression pattern both in the central nervous system and the body. The receptor has been investigated as a potential target for a multitude of significant diseases including migraine, alcohol use disorder, ischemia, and neurodegenerative diseases. Despite multiple attempts, delta opioid receptor-selective molecules have not been translated into the clinic. Yet, the therapeutic promise of the delta opioid receptor remains and thus there is a need to identify novel delta opioid receptor ligands to be optimized and selected for clinical trials. Here, we highlight recent developments involving the delta opioid receptor, the closely related mu and kappa opioid receptors, and in the broader area of the GPCR drug discovery research. We focus on the validity and utility of the available delta opioid receptor structures. We also discuss the increased ability to perform ultra-large-scale docking studies on GPCRs, the rise in high-resolution cryo-EM structures, and the increased prevalence of machine learning and artificial intelligence in drug discovery. Overall, we pose that there are multiple opportunities to enable in silico drug discovery at the delta opioid receptor to identify novel delta opioid modulators potentially with unique pharmacological properties, such as biased signaling.

## 1. Introduction

The δ opioid receptor (δOR) is a Gi-protein-coupled receptor with a broad expression pattern both in the central nervous system and the periphery. The endogenous agonists for the δOR are pentapeptide enkephalins, particularly Leu^5^-enkephalin, but other peptides that originate from plants and other species, like frogs can also bind and activate the δOR [1,2]. Similar to the µ opioid receptor (µOR), which is activated by small molecules from natural products like opium and kratom and fully synthetic small molecules like fentanyl, the δOR can be activated by a variety of naturally occurring and (semi-) synthetic small molecules [3,4,5,6].

The δOR has been a potential candidate to treat a variety of diseases and disorders. Front and center have been the ability of δOR selective agonists to reduce chronic pain, be it inflammatory, neuropathic, or migraine [7]. δOR agonists have shown promise in preventing cardiac and cerebral ischemia [8], as a potential treatment for neurodegenerative diseases [9,10,11,12,13], and both δOR agonists and antagonists have been proposed as mechanisms for the treatment of alcohol use disorder [5,14,15,16]. Outside the central nervous system, δOR antagonism and positive allosteric modulation of δOR has been proposed as a treatment for gastrointestinal motility disorders, such as irritable bowel syndrome [17,18].

Early attempts by SmithKline Beecham to synthesize δOR agonists to suppress cough (SB 227122), or to treat inflammatory pain without causing seizure activity (SB 235863) [19,20,21] did not progress to clinical trials. While δOR-selective agonists, including ADL5859 and AZD2327, have previously entered clinical trials for the treatment of pain and depression, no δOR selective drugs have ultimately been approved for human use. [22,23,24] Both ADL5859 and AZD2327 failed to advance beyond phase II clinical trials, which are designed to establish efficacy in patients within the therapeutic indication space. ADL5859 and AZD2327 are part of a class of diethylbenzamides that include the prototypical δOR-selective agonist SNC80. However, SNC80 and multiple other δOR agonists have been reported to reduce seizure threshold and induce convulsions [25,26,27], and this has reduced enthusiasm for δOR agonists as a therapeutic area of research.

Around the same time that ADL5859 and AZD2327 were in clinical trials, Johnson and Johnson developed the anti-hyperalgesic δOR agonists, JNJ-20788560 and RWJ-394674 [28,29], but they did not take these into clinical trials, potentially due to the failure of the aforementioned clinical trial compounds. Prior to becoming insolvent, Ardent Pharmaceuticals, also produced multiple δOR agonists, with mixed µOR activity in their DPI series (DPI-221, DPI-125, DPI-289) in hopes of producing an analgesic drug with fewer adverse effect liabilities relative to the clinically used µOR agonists [12,30,31].

Recent studies suggest that β-arrestins, multifunctional proteins that can promote receptor desensitization and intracellular signaling, are involved in the mechanism of seizure activity of SNC80 [26]. This insight has spurt efforts to develop G-protein-biased δOR agonists to reduce adverse effects including seizures, paralleling similar efforts for increasing the therapeutic window through G-protein-biased agonism at other GPCRs, including the µOR. These endeavors have generated a multitude of peptides with reduced β-arrestin recruitment potency/efficacy [3,32,33,34,35]. Similarly, small molecule biased agonists have also been developed including TAN-67, KNT-127, TRV250, and most recently PN6047. Indeed, these G-protein-biased δOR agonists appear to suffer less from detrimental side effects including no seizure activity, no hyperlocomotion, and no rewarding effect [36,37,38,39]. Positive and negative allosteric modulators (PAM, NAM) and bitopic opioids that act as ‘Ago-PAM’ or ‘Ago-NAM’ have been identified and modeled in the δOR binding pocket. The benefit of pure allosteric modulators is that they are inert in the absence of enkephalin and only amplify (PAM) or inhibit (NAM) δOR signaling and/or binding when, for example, enkephalins are synaptically released. Allosteric modulators may promote a particular signaling conformation and bias the endogenous peptide; such a strategy could reduce the risk of tachyphylaxis, off-target effects, and even on-target side effects. Less than ten years ago now, Bristol Meyers Squibb identified a number of µOR and δOR Ago-PAMs [40,41,42] and more recently, novel δOR agonists lacking a basic nitrogen, including a novel chemotype and bitopic ligand, were also identified [43,44]. As these molecules are very recent their clinical utility has not been explored in much depth, but δOR PAM activity may, for example, aid the treatment of irritable bowel syndrome [18].

Still, except for TRV250, which has undergone phase I clinical trials for migraine, no real progress has occurred towards the production of δOR-based clinical candidates. Thus, there both remains a need and a large opportunity for discovering and developing novel δOR agonists as potential therapeutic agents. Here, we present a summary of current structural data that has been generated for the δOR; we first will provide an overview of available resolved structures and insights gained from mutagenesis studies and MD simulations. We will then discuss the limitations of the current structural knowledge. We conclude this review by presenting exciting opportunities for computer-aided drug design at the δOR.

## 2. Current Structural Insight in δOR Binding Pocket and Activation Mechanism

Early mutagenesis studies following the cloning of the δOR [45,46] hypothesized the involvement of certain amino acids in ligand recognition, selectivity, and overall receptor activation. One of the earliest mutagenesis studies investigated the role of Asp2.50 (Ballesteros-Weinstein numbering, [47]) in regulating ligand binding at the δOR where they demonstrated that Asp2.50Asn diminishes binding of peptide agonists with minimal effects on alkaloid agonists and antagonist [48]. Additionally, the authors hypothesized that Asp2.50 is in close proximity to the Na^+^ binding site [48]. This was followed by another study, where Asp3.32 was investigated due to its conservation across many GPCRs that are activated via cationic neurotransmitters (protonated amines) [49]. The removal or replacement of that residue affects the binding of several δOR modulators. Unlike the alanine scanning mutations, the replacement of Asp3.32 with Asn3.32 resulted in modifications to the receptor’s pharmacology and affected the binding potency of alkaloid and peptide-agonists. The authors hypothesized that the main reason for such dramatic change may be attributed to the increase in the size of the Asn3.32 side chain compared to the WT residue. Asp3.32 acts as a proton donor only whereas Asn3.32 can act as a hydrogen donor or acceptor. Additionally, the authors noted that the “Na^+^ -induced low-affinity state” lowered the affinity of δOR peptide agonists such as DTLET and DADLE but did not affect the binding affinity of SNC-80 or BW373U86 (SNC-86) in agreement with the work by Kong et al. [48]. Interestingly, when the authors tested the δOR agonists DTLET, DADLE, and SNC-86 in the presence of sodium chloride, the binding affinity was reduced dramatically indicating a role for Asp2.50 in receptor activation to counteract the negative allosteric effects of the Na^+^.

The same group later used single-point mutagenesis to investigate the involvement of aromatic amino acid residues (Tyr, Trp, and Phe) in transmembrane helices III–VI in ligand recognition [50]. To identify which aromatic residues to target, they used computational modeling to construct a 3D homology model for the δOR based on the human rhodopsin and hamster β2-adrenergic receptors. They showed that mutations Tyr mutations (Tyr3.33, Tyr7.42) had the most impact on the binding of deltorphin II [50]. They concluded that each ligand-receptor complex has unique binding and conformation where mutations do not have the same effect equally across various δOR ligands [50].

Another research team created a chimeric protein, DMDD, by replacing the area around the 1st extra-cellular loop 1 (ECL1) with the corresponding residues of µOR which significantly enhanced DAMGO binding to δOR [51]. A subsequent study replaced seven non-conserved residues in transmembrane domain (TM2) and TM3 with the corresponding residues in µOR and found that one residue only, Lys2.63 (replaced by Asn2.63) showed a high affinity for DAMGO. Replacement of Lys2.63 with nineteen different amino acid residues resulted in fourteen mutant receptors that could bind to DAMGO with comparable affinity to the DMDD chimera indicating a role for Lys2.63 to act as a recognition switch for δOR agonists [52]. A similar approach of using chimeric constructs for δOR in a different study demonstrated the importance of the ECL3 in the binding of selective peptide and small molecule agonists to the δOR. In the same study, the authors showed that three residues, Trp6.58, Val7.30, and Val7.31 are necessary for the binding of δOR agonists [53].

These mutagenesis studies have provided valuable insight into ligand recognition and receptor selectivity of the δOR some of which have been verified in recent structural studies (discussed below). However, these studies did not provide insight into the effect that the investigated mutations have on downstream signaling cascades at δOR. More importantly, there is a gap in knowledge with respect to the impact of most of these mutations on biased agonism.

**Figure 1:**
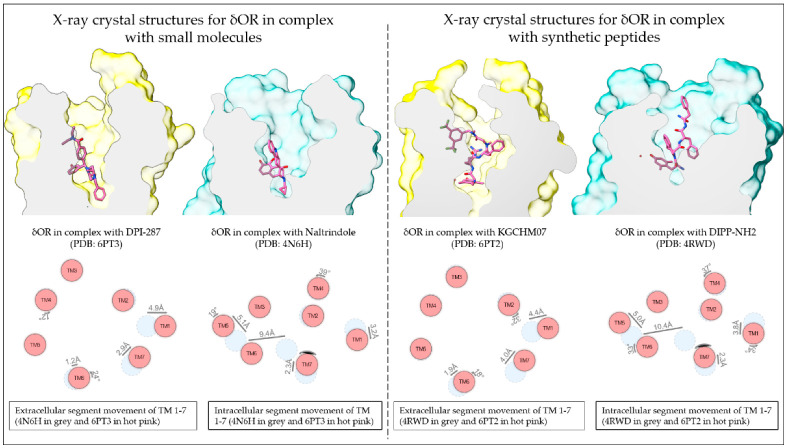
Resolved structures of the δOR in complex with small molecules and peptides. Schematic depiction of the small molecule agonist DPI-287 and antagonist naltrindole and the peptide agonist KGCHM07 and antagonist DIPP-NH2 bound to the δOR (Top panels; active-like structures in yellow and inactive structures in sea green). The difference in TM domain positions between the antagonist- and agonist-bound structures (Lower panels; antagonist-bound in grey and agonist bound in hot pink). TM domain positions produced using the structure comparison tool from GPCRdb.

**Figure 2:**
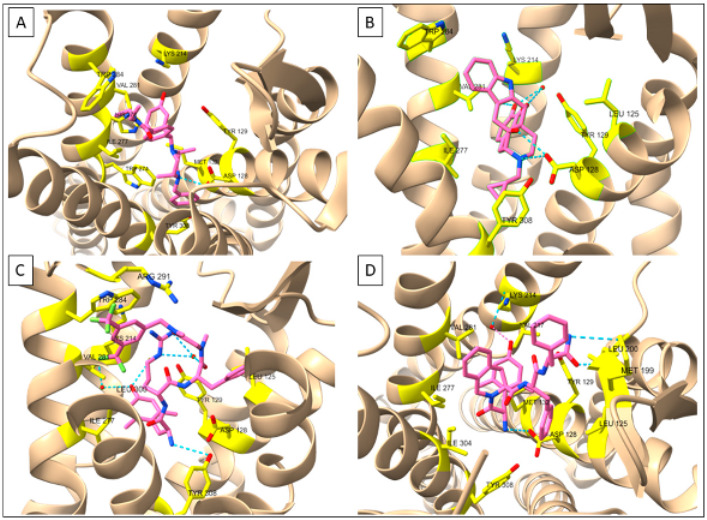
Receptor-ligand interactions at δOR deduced by X-ray crystallography. (**A**) δOR-DPI287 (PDB: 6PT3) (**B**) δOR-NTI (PDB: 4N6H (**C**) δOR-KGCHM07 (PDB: 6PT2) (**D**) δOR-DIPP-NH2 (PDB: 4RWD). Figures made in ChimeraX 1.1.

Over the past decade, several moderate to high-resolution structures of the δOR have been produced and have confirmed older evaluations of the δOR binding pocket performed by mutagenesis and computational modeling (Figure 1, Table 1) [49,54,55]. In the first such structure (3.4Å, PDB:4EJ4), δOR was bound to the antagonist naltrindole [56]. The structure confirmed the important interaction between Asp3.32 with the protonated amine of naltrindole, which mimics the amine of Tyr^1^ in endogenous OR peptides. As mentioned, this Asp3.32 was already known to be crucial for opioid affinity from mutagenesis studies [49]. In this structure, anchors are provided by hydrogen bonds (potentially including water molecules) with His6.52 and Tyr3.33 [56]. Additional amino acids surrounding the binding pocket were Met3.36, Trp6.48, Ile6.51, Val6.55, Trp6.58, Leu7.35, and Tyr7.43 [56]. A higher resolution structure (1.8Å, PDB:4N6H) of naltrindole-bound δOR was able to resolve an allosteric binding site for a sodium ion (Figure 2B) [57]. The sodium site consists of Asp2.50, Asn3.35, Asn7.45, and Asn7.49 and sits below the conserved ‘message’ site [‘message’ = part of the molecule that recognizes ORs, ‘address’ = part that renders the drug subtype selective] of the opioid receptor binding pocket [58]. In 2015, a third δOR structure (2.7Å, PDB:4RWD) was resolved, but this time bound to the δOR peptide antagonist Dmt^1^-Tic^2^-Phe^3^-Phe^4^ (DIPP-NH2, Dmt = 2,6,-dimethyl-l-tyrosine) (Figure 2D) [59].

The DIPP-NH2 binding pocket utilized the same ‘message’ binding pocket residues as naltrindole with slight movements of Val6.55 and Trp6.58. As expected, the Dmt^1^ residue interacted with Tyr3.33, Ile6.51, and Val6.55 with the N-terminal amine forming a salt bridge with Asp3.32 [57]. The Tic^2^ side group resided in a hydrophobic pocket made up of Ile6.51, Val6.55, Trp6.58, Leu7.35, and Ile7.39. Importantly, the larger surface of DIPP-NH2 extended further into the ‘address’ portion of the δOR binding pocket, with Phe^3^ interacting with Leu3.29, Asp3.32, and Tyr3.33. Phe^4^ interacted with a Met and Leu in ECL2. In the µOR, the corresponding amino acids are charged/polar, making this a possible region important for selectivity [59,60] (Table 2).

In 2019, two agonist-bound X-ray crystal structures produced novel insight into δOR in the active-like state: the δOR bound to the peptide KGCHM07 (PDB:6PT2) and bound to the SNC80-like small molecule DPI-287 (PDB:6PT3) [61] (Figure 2A,C). Relative to the naltrindole-bound structure, the agonist structure shows the movement of TM6 (Figure 1 and Figure 2), particularly, Phe6.44, Cys6.47, and Trp6.58, all of which had been previously linked to δOR activation [55,61]. Arg291 in ECL3 changes location in the KGCHM07 structure and forms a lid on the binding pocket and is part of a hydrophobic pocket that also includes, Ile6.51, Phe6.54, Val6.55, Trp6.58, and Leu7.35, which fits the benzyl moiety of KGCHM07 (Figure 2). Three water molecules interacted with Dmt^1^ of KGCHM07 through Tyr3.33, Lys5.39 and His6.52. Water molecules also aid in forming a water-mediated salt-bridge between D-Arg^2^ and Asp5.35. Slight differences were observed between the peptide and small molecule structures particularly in relation to the polar network, involving Thr2.56, Glu2.60, and Tyr7.43 the latter being part of a hydrophobic pocket that fits Phe^3^ in the peptide-bound structure. Tyr7.43 stabilizes the primary amine of KGCHM07 but does not interact with DPI-287. On the other hand, Thr2.56 stabilizes the polar network for DPI-287 but not KGCHM07 [61]. Overall, the antagonist structures confirmed many predicted and experimentally established key amino acids within the δOR binding pocket. Paired with the addition of the agonist-bound structures this provides new avenues and opportunities for in silico drug discovery at the δOR.

## 3. Limitations of Current δOR Structures

All the current δOR structures have been resolved using X-ray crystallography (Table 1). The nature of X-ray crystallography relies heavily on producing a receptor that is stable and does not show a lot of movement. On the other hand, cryo-electron microscopy (cryo-EM) is more forgiving in this regard, and thereby provides more opportunities to generate a structure of a wild-type/non-thermostabilized receptor to overcome the current hurdle that the available δOR agonist structures are mutated. Importantly, the Sexton group at Monash University has made significant improvements in the workflow for generating cryo-EM structures, such that structures with resolutions below 3Å can now be routinely resolved [64].

Another limitation for in silico drug discovery at the δOR is that none of the δOR structures were co-crystallized with an effector protein. The ability of cryo-EM to determine the structures of large complexes of macromolecules gained attraction over the past decade following advancements in electron detectors and data software used to reconstruct the 3D structures from the 2D images [64]. For GPCRs in complex with downstream effector proteins, such as G-protein, β-arrestin, or GRKs, cryo-EM is increasingly becoming the method of choice for structure determination. This is in part because protein structures obtained using cryo-EM overcome some of the limitations such as thermostabilizing mutations and fusion proteins which are commonly introduced in X-ray crystallography structures [65]. The increasing number of cryo-EM structures that are being obtained in complex with downstream effector proteins can provide valuable insight into the molecular basis of GPCR signaling, potentially biased signaling, which then informs the structure-based drug discovery process.

Thus far, three cryo-EM structures of the µOR and one structure for the κ-opioid receptor (κOR) have been resolved [66,67,68]. Nonetheless, with respect to the δOR, the absence of X-ray crystal structures or cryo-EM structure of the δOR in complex with downstream effector proteins represents a challenge for structure-based drug discovery. Overcoming this hurdle requires careful and extensive molecular modeling that integrates the available crystal structures of the δOR in their inactive- and active-like states (Table 1) with the structures of other opioid receptors that are in complex with Gi-proteins, β-arrestins, nanobodies. This is especially crucial when using the active-like crystal structures of δOR due to the presence of thermostabilizing mutations. This method of computational structure determination was applied at the κOR where molecular dynamics (MD) simulations and an enhanced sampling method called meta-dynamics simulations were used to determine the structure of the κOR in complex with the Gi-protein. To obtain the optimized active structure, the authors started with optimizing the nb39 stabilized crystal structure of the κOR in complex with the agonist MP1104 (PDB: 6B73) then used the µOR-DAMGO-nucleotide free Gi-protein cryo-EM structure to couple the Gi-protein to the κOR [66,67,69]. Then, they used meta-dynamics simulations to optimize the κOR-Gi complex interactions before examining its stability using MD simulations. Their approach was applicable to the crystal structure of the µOR (5C1M) which they used to construct µOR-BU72-Gi and confirm the structural determinants of G-protein coupling.

To obtain the active-like δOR x-ray crystal structures bound to a peptide agonist and to a small molecule agonist, the δOR was thermostabilized by nine point mutations that negatively impacted the native pharmacology [61]. This was unsurprising, as the impact of mutations on receptor function can compound with increasing numbers [55]. Particularly, the mutations impacted the allosteric sodium binding pocket that has been implicated in β-arrestin signaling [57,61]. Thus, the crystallized conformation may not be optimal to identify signal-biased agonists reducing the utility of the current agonist-bound δOR structures.

## 4. Opportunities for Computer-Aided Drug Discovery at the δOR

Over the last ten years, an increasing number of opioid receptors structures have been elucidated in unbound (apo-state), antagonist bound, or agonist bound states, either stabilized with thermostabilizing mutations, G_i_-protein, or nanobodies. These structures, even the antagonist-bound ones, have proven useful for performing docking studies on large virtual libraries. For example, a screen of 3M molecules on the inactive µOR (4DKL) led to the identification of a hit that was optimized in three steps to the novel G-biased agonist PZM21 [70]. Recent advances in docking have enabled the screening of libraries of nearly two magnitudes larger in size; thus far, this approach has been successfully employed to screen 138 million compounds using an antagonist-bound dopamine D_4_ receptor [71], and 150 million compounds at a thermostabilized agonist-bound melatonin MT1 receptor [72]. These screens relied on the ZINC database [73] to provide decoys and screening molecules. The ZINC database is an ever-increasing repository for accessible molecules, currently holding about one billion compounds [74,75]. The increase in the size of the ZINC database is largely supported by the increase in catalog size of commercially available compounds from Enamine (https://enamine.net/news-events/press-releases/807-enamine-expands-collaboration-with-ucsf, accessed on 12 July 2022). Indeed, commercially available make-on demand chemical libraries such as Enamine’s REAL Space collection which comprises 22.7 billion compounds as of the time of writing this review, make it feasible to identify novel chemotypes that could induce novel pharmacology. It comes as no surprise that high throughput virtual screening campaigns are expected to further expand the utilized ligand chemical space which will result in the identification of an increasing number of novel hit compounds. This expansion in the utilized chemical space can be effectively leveraged for structure-based drug discovery using currently available, and future, structures of δOR to virtually dock and screen more compounds than ever before.

**Figure 3:**
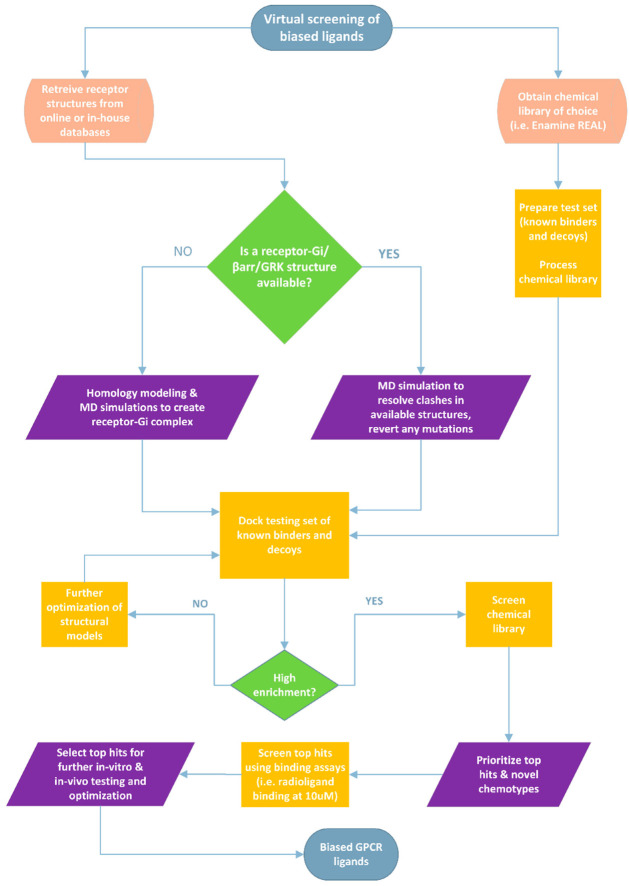
Proposed workflow for screening large chemical libraries to identify G-protein biased agonists at the δOR and other GPCRs. A similar workflow could be applied to identify GRK- or β-arrestin-biased small molecules given that high-quality crystal or cryo-EM structures are available. In cases where distinct interactions or sub-pockets specific to biased agonists or in cases where we know that an allosteric site/pocket could lead to biased effects, we could restrict ligand docking to that specific site to screen a given chemical library. The most accurate way to confirm such interactions would be to resolve high-quality structures and/or perform mutagenesis studies. Alternatively, enhanced sampling computational modeling to model receptor-effector complexes could be useful if computational cost is not a limiting factor.

Efforts in virtual screening of chemical libraries have used structure-based drug discovery to expand the available chemical space for various GPCR targets [72,76]. This approach has yielded many novel chemotypes across several targets that could provide useful starting points for medicinal chemists to modify and improve selectivity among other properties. However, in screening campaigns that aim to identify biased agonists in-silico, this approach represents a potential bottleneck to the discovery process and might not be sufficient to identify functionally selective ligands. Thus far, the method of choice in identifying biased agonists at the δOR and other GPCRs has relied on the functional characterization of known and novel binders using cell-based assays to establish pharmacological and SAR profiles for future hit identification and lead optimization campaigns. However, the recent advances in computational tools and enhanced sampling methods such as molecular dynamics simulations which enable the dynamic modeling of the δOR and other GPCRs should be leveraged to unravel the structural determinants of biased signaling. In other words, the structural and conformational changes induced by agonist binding at δOR that appear in an MD simulation could be correlated with pharmacological data and mutagenesis analyses. Such an approach could be used to generate structural models or snapshots of the δOR in different conformational states which would increase the ability of docking campaigns to identify novel and biased agonists (Figure 3).

Until recently, GPCRs’ structures in general, including those of the opioid receptors, still do not appear to provide a clear picture of the underlying signaling mechanisms given the complexity of the involved signaling network, the limited number of available structures bound to G-proteins, β-arrestins, or GRKs, and the diversity of the chemical space interacting with these receptors [77]. The tremendous success of cryo-EM structure determination and the increasing number of high-resolution structures when coupled with MD simulations could provide insights into GPCRs in action. Hence, developing and utilizing computational methods and workflows to model ensembles of structural conformations and then combining such methods with resolving GPCR structures in complex with G-proteins, β-arrestins, and GRKs should provide a strong approach to alleviate current limitations. This could also minimize the misinterpretations that stem from comparisons and analyses that are based on static GPCR structures that suffer from the limitations mentioned above.

A similar approach was applied recently at the µOR, where Wang et al. highlighted how the implementation of molecular dynamics could be helpful in lead optimization campaigns [68]. The authors implemented a structure-based lead optimization approach to generate PZM21 analogs with improved CNS penetration and higher G-protein bias with lower β-arrestin recruitment compared to fentanyl. In the study, the authors resolved a high-resolution cryo-EM structure of PZM21 bound to the μOR in a complex with the trimeric Gi-protein (PDB: 7SBF). The resolved cryo-EM structure showed that PZM21 forms a salt bridge between its basic amine and Asp3.32 of μOR which confirmed previous findings [67,78]. To confirm the stability of the PZM21′s binding pose, and characterize the water-mediated interactions with μOR, the authors performed all-atom MD simulations which showed that PZM21 formed water-mediated interactions with His6.52 and Lys5.39. Intriguingly, the authors used insights from structural and dynamic analyses for lead optimization, guided by MD simulations and the cryo-EM structure of μOR in complex with Gi-protein. The resulting PZM21 analogs were μOR-selective with improved functional selectivity. Translating such an approach at the δOR will allow for more successful hit-to-lead and lead-optimization campaigns. Recently resolved cryo-EM structures of mitragynine pseudoindoxyl, which has very low arrestin recruitment, and lofentanil, a potent arrestin recruiter demonstrate the involvement of distinct orthosteric sub-pockets in determining arrestin recruitment at the μOR. The authors demonstrated that each agonist has distinct moieties that bind in two distinct sub-pockets while sharing a central binding pocket with DAMGO [79]. Such findings support previous predictions and lead optimization strategies that have been applied to modulate biased agonism at the μOR and κOR [80,81].

Another promising area that has been on the rise recently is using machine learning and artificial intelligence in protein structure prediction and drug discovery. These advances present an exciting avenue for the discovery of novel and potential therapeutic agents at the δOR. Undeniably, the integration of machine learning and deep learning with current computational and pharmacological approaches presents a valuable opportunity to accelerate drug discovery campaigns at δOR. Machine learning models could be trained using high-quality datasets to predict drug properties, toxicity, target selectivity, and potentially ligand-receptor interactions. This has been made possible in part due to the GPCR community’s efforts to provide access to curated datasets such as GPCRdb.org [62,82] and open-source machine learning packages such as DeepChem and AMPL, which allows researchers to build, train and deploy machine learning models for drug discovery [83]. Additionally, the rapid increase in high-performance cloud computing, improved Graphics Processing Units (i.e., GPUs), and increasingly efficient machine learning algorithms provide an opportunity to expand the screening of ultra-large chemical libraries or the de-novo drug design in a more efficient manner.

The significant improvement in structure prediction provided by AlphaFold 2 [84], may provide avenues for obtaining a wild-type thermostable δOR structure, that could be used for docking studies. There are significant limitations, in particular the current lack of Alphafold to predict how a protein will change conformation upon binding of a specific ligand [85]. Future machine learning algorithms may learn how to do this and further reduce the initial requirement of wet-lab science to obtain potent and selective novel molecules for a receptor target including the δOR.

MD simulations are another area that is expected to benefit from recent advances in machine learning and artificial intelligence. In fact, work is already underway to develop machine learning force fields that could increase the accuracy of MD simulations while reducing the computational cost [86]. Deep learning frameworks such as TorchMD [87] and neural networks such as graph convolutional neural networks (GCNNs) have been used for geometry optimization [88], acceleration of MD simulations, or even in improving force fields [89]. Such advances should be carefully utilized and expanded to complement the available structural and experimental data and accelerate the identification of therapeutic agents at the δOR.

To accelerate future large-scale drug discovery efforts at the δOR, well-trained and validated machine learning models should be combined with physics-based scoring functions to reduce the computational cost of docking and screening ultra-large chemical libraries. In this instance, a machine learning model could serve as a filter that could be incorporated into a docking workflow to prioritize which molecules move on to the next phase and ultimately which molecules are to be tested pharmacologically. Moreover, this approach allows the incorporation of structural and pharmacological parameters to build, train, and deploy multi-task learning models that could increase the selectivity and novelty of the identified compounds.

Despite the optimistic outlook and promising advances in computer-aided drug design, it is important to know the limits of the utilized tools. Furthermore, it is worth noting that there are numerous challenges and potential pitfalls associated with the incorporation of machine learning or deep learning models, especially with respect to the availability of large and high-quality datasets for the drug target [90,91]. For the δOR and other GPCRs, the diversity of cell-based assays that are used to characterize their pharmacology and the inconsistent practices between different research labs, or in some instances within the same lab, are two major limitations that need to be addressed before we can reliably use machine learning in GPCR drug discovery. Hence, future efforts should also focus on the standardization of experimental data collection and computational data curation and modeling.

## 5. Conclusions

For the identification of novel, functionally selective, and potentially therapeutic agonists at δOR, future efforts should aim to expand our understanding of the effect of various mutations on the structure and function of δOR utilizing the constantly improving *in-vitro* and *in-silico* approach. Additionally, producing multiple wild-type agonist-bound high-resolution structures of δOR will allow for a more efficient expansion of its chemical space in virtual screening campaigns or in computer-aided lead optimization. Consequently, these efforts will provide high-quality datasets that could allow for the incorporation of ML and DL tools in opioid drug discovery. Overall, we think there are more opportunities than that there are challenges to carry out a high yield in silico screen at the δOR to generate novel chemical matter that hopefully can be translated into meaningful therapeutics.

## Figures and Tables

**Table 1:** Overview of resolved x-ray crystal structures of the δOR. Table produced using the GPCRdb.

Structure						Auxiliary Protein	Structure Ligand		
Method	PDB	Resolution	State	Degree Active (%)	% of Seq	Fusion	Name	Type	Function
X-ray	6PT2	2.8	Active	76	78	BRIL	KGCHM07	peptide	Agonist
X-ray	6PT3	3.3	Active	76	78	BRIL	DPI-287	small-molecule	Agonist
X-ray *	4RWD	2.7	Inactive	7	79	BRIL	DIPP-NH2	peptide	Antagonist
X-ray	4RWA	3.3	Inactive	7	77	BRIL	DIPP-NH2	peptide	Antagonist
X-ray	4N6H	1.8	Inactive	7	81	BRIL	Naltrindole	small-molecule	Antagonist
X-ray	4EJ4	3.4	Inactive	7	76	T4-Lysozyme	Naltrindole	small-molecule	Antagonist

* 4RWD structure was obtained using the XFEL method.

**Table 2:** Receptor-ligand interactions of the δOR in complex with peptide and small-molecule agonists and antagonists. Table produced in part using the GPCRdb. [62,63]. This table does not reflect the full extent of receptor-ligand interactions, especially with regards to the involvement of the amino acid residues forming hydrophobic sub-pockets of the orthosteric site that are necessary for ligand binding. Additional amino acid residues such as Asp2.50, Asn3.35, and Ser3.39 which form the sodium binding site are also not included in this table.

				Agonist		Antagonist			
				6PT2	6PT3	4RWD	4RWA	4N6H	4EJ4
Amino Acid	Sequence Number	Generic Number	Segment	KGCHM07	DPI-287	DIPP-NH2		Naltrindole	
A	98	2.53	TM2						
L	125	3.29	TM3						
D	128	3.32	TM3						
Y	129	3.33	TM3						
M	132	3.36	TM3						
M	199	ECL2	ECL2						
L	200	ECL2	ECL2						
D	210	5.35	TM5						
K	214	5.39	TM5						
V	217	5.42	TM5						
W	274	6.48	TM6						
I	277	6.51	TM6						
H	278	6.52	TM6						
V	281	6.55	TM6						
W	284	6.58	TM6						
R	291	ECL3	ECL3						
L	300	7.35	TM7						
I	304	7.39	TM7						
Y	308	7.43	TM7						
**Color legend:**		Hydrophobic		Aromatic (face to edge)		Aromatic (face to face)		Accessible	
polar (charge-assisted hydrogen bond)			polar (charge-charge)		polar (hydrogen bond)		polar (hydrogen bond with backbone)		

## Data Availability

Not applicable.
